# Perceptions and Intentions to Use Generative AI Among First-Year Medical Students in Japan: Cross-Sectional Survey Study

**DOI:** 10.2196/77552

**Published:** 2025-11-13

**Authors:** Hiroshi Tajima, Hajime Kasai, Kiyoshi Shikino, Ikuo Shimizu, Shoichi Ito

**Affiliations:** 1 Department of Medical Education, Graduate School of Medicine, Chiba University, 1-8-1 Inohana, Chuo-ku, Chiba, 260-8670, Japan, 81 43-222-7171 ext 73320, 81 43-311-3614; 2 Health Professional Development Center, Chiba University Hospital, Chiba, Japan; 3 Department of Community-Oriented Medical Education, Graduate School of Medicine, Chiba University, Chiba, Japan

**Keywords:** generative artificial intelligence, medical students, digital literacy, perceptions, learning behavior, Japan

## Abstract

An April 2025 survey of 118 first-year Japanese medical students found high use of generative artificial intelligence (84.7%) but limited formal learning (49.2%), with strong learning interest yet neutral assignment use, indicating a need for structured literacy in generative artificial intelligence.

## Introduction

Generative artificial intelligence (GenAI), particularly large language models like ChatGPT (OpenAI), has emerged as a tool for brainstorming, information gathering, proofreading, translating, and other academic tasks [1]. The rapid evolution of ChatGPT has made it essential for medical students to understand and use these tools [2]. As digital technologies become more embedded in health care education, understanding how future physicians will engage GenAI is vital. Many students demonstrate familiarity with digital tools and are exposed to them during childhood. One study showed that medical students had positive attitudes toward GenAI, albeit with low use rates [3]. In Japan, although some educators have adopted GenAI, information on how incoming medical students educated during the GenAI boom perceive these tools remains limited [4]. Thus, we explored GenAI use, learning behaviors, and perceptions of first-year medical students in Japan.

## Methods

### Overview

An anonymous online survey targeting first-year students immediately after high school graduation was conducted at the Chiba University School of Medicine in April 2025; 120 students were invited to participate and 118 valid responses were received (response rate 98.3%).

The questionnaire comprised four sections: (1) demographics; (2) prior GenAI exposure; (3) willingness to learn and perceptions (16 items rated on a 5-point Likert scale [1=strongly disagree to 5=strongly agree], based on prior studies [5,6]); and (4) an open-ended item on academic use. The items were pilot-tested for clarity and reliability.

Group differences were examined using independent 2-tailed *t* tests and chi-square tests. Spearman correlations were used to identify factors associated with learning motivation. Internal consistency (Cronbach α) was 0.81 for risk awareness (6 items), 0.81 for benefit recognition (6 items), and 0.53 for concern items (4 items; reverse-coded). Statistical significance was set at *P*<.05. Two independent coders performed content analysis of open-ended responses, with substantial agreement (κ=0.67). Analyses were conducted using JMP Pro (version 18; JMP Statistical Discovery LLC).

### Ethical Considerations

This study was approved by the ethics committee of Chiba University Graduate School of Medicine (approval 3425). Electronic informed consent was obtained prior to participation. The study database was anonymized. Study data are stored on an offline, password-protected computer with full-disk encryption. Participants received no compensation for this study. All methods were in accordance with the relevant guidelines and regulations.

## Results

Among 118 responses (n=79, 66.9% male; mean age 18.5, SD 0.7 years), 84.7% (n=100) reported GenAI use, mainly for language learning (n=53, 44.9%) and information searches (n=46, 39.0%). Only 49.2% (n=58) of the participants had learned about GenAI, primarily through browsing (n=34, 28.8%) or through peers (n=24, 20.3%).

Students demonstrated high willingness to learn (mean score 4.3, SD 0.9), with good understanding (mean scores 3.9‐4.6) and positive attitudes, especially regarding the relevance of GenAI (mean score 4.3, SD 0.8) (Table 1). Perceived individualization was lower (mean score 3.5, SD 1.0), with concerns over educational impact (mean score 3.6, SD 0.9).

Willingness to learn correlated positively with expectations of GenAI’s benefits, such as enhancing digital literacy and career preparedness (both *P*<.001).

Students with learning experience were slightly younger (mean age 18.4, SD 0.7 vs 18.7, SD 0.8 years; *P*=.02) and demonstrated greater awareness of potential bias in GenAI outputs (*P*=.045).

**Table 1.** Medical students’ responses regarding demographics, knowledge, willingness to use, and concerns about generative artificial intelligence (GenAI) technologies.

Item		All (n=118)	Experienced^a^ group (n=58)	Inexperienced group (n=60)	*P* value
Sex, n (%)					.13
	Male	79	35	44	
	Female	39	23	16	
Age (years), mean (SD)		18.5 (0.7)	18.4 (0.7)	18.7 (0.8)	.02
Willingness to learn about GenAI (1=strongly negative to 5=strongly positive), mean score (SD)		4.3 (0.9)	4.3 (0.8)	4.2 (0.9)	.32
Attitude toward using GenAI for assignments (1=strongly negative to 5=strongly positive), mean score (SD)		3.0 (1.0)	2.9 (1.0)	3.0 (1.0)	.65
Knowledge of GenAI technologies, mean score (SD)					
	I understand that GenAI has limitations in handling complex tasks.	3.9 (0.9)	4.1 (0.9)	3.9 (0.9)	.21
	I understand that GenAI may produce outputs that are factually incorrect.	4.6 (0.7)	4.6 (0.6)	4.6 (0.7)	.67
	I understand that GenAI may generate outputs that are contextually inappropriate.	4.4 (0.7)	4.4 (0.7)	4.4 (0.6)	.9
	I understand that GenAI outputs may sometimes reflect bias or discrimination.	4.0 (0.9)	4.2 (0.9)	3.9 (0.9)	.04
	I understand that GenAI is pattern-based and may have limited applicability in specific contexts.	4.0 (1.0)	4.1 (1.0)	3.8 (0.9)	.11
	I understand that that GenAI lacks emotional intelligence and may produce insensitive content.	4.1 (1.0)	4.2 (0.9)	3.9 (1.0)	.11
Willingness to use GenAI technologies, mean score (SD)					
	I am considering incorporating GenAI into my learning and practice in the future.	3.9 (0.8)	3.9 (0.8)	3.8 (0.9)	.53
	Students need to learn how to effectively leverage GenAI for their career paths.	4.3 (0.8)	4.3 (0.7)	4.3 (0.9)	.59
	I believe that GenAI can enhance my digital literacy skills.	3.7 (1.0)	3.8 (0.9)	3.6 (1.1)	.33
	I believe GenAI can offer novel insights beyond my own thinking.	3.8 (0.9)	3.8 (0.8)	3.9 (0.9)	.73
	I believe that GenAI can instantly offer personalized suggestions for my assignments.	3.5 (1.0)	3.5 (1.0)	3.5 (1.0)	.99
	I think GenAI is a great tool because it is available 24-7.	4.0 (0.9)	4.0 (0.8)	3.9 (1.0)	.36
Concerns about GenAI technologies, mean score (SD)					
	Completing assignments using GenAI undermines the value of a university education.	3.6 (0.9)	3.6 (0.9)	3.5 (0.9)	.53
	GenAI limits opportunities for students to interact during face-to-face lectures and group work.	3.2 (1.0)	3.3 (0.9)	3.1 (1.0)	.25
	GenAI hinders the development of portable skills, such as teamwork, problem-solving, and leadership.	3.5 (0.9)	3.6 (0.8)	3.4 (1.0)	.12
	I feel that I am becoming overly dependent on GenAI.	2.1 (1.1)	2.2 (1.2)	2.0 (1.1)	.25

a “Experienced” refers to students who reported any learning about GenAI, including formal instruction and learning from informal sources, such as web browsing or peers.

The attitude toward using GenAI for assignments was neutral (mean score 3.0, SD 1.0). Open-ended responses were categorized into 3 attitudinal groups, “positive,” “cautious,” and “negative,” reflecting enthusiasm, balanced concerns, or skepticism (Table 2).

**Table 2.** Keyword categories extracted from free-text responses regarding the academic use of generative artificial intelligence (GenAI).

Category		Keywords	Responses, n	Quotes
Positive				
	Convenience and efficiency	Efficient, convenient, easy	25	GenAI improves work efficiency because it can quickly search for information.
	Information handling and use	Information gathering, use, referencing, critical thinking	23	Using GenAI as a mock interviewer to question my responses was helpful.
	Quality and outcomes	Quality improvement, attractive, useful	9	High-quality information can be obtained.
	Necessity and attitude	Necessary, available resources	6	We should develop the ability to use GenAI effectively.
	Creativity	Original, idea	4	By integrating it with my own ideas, I can come up with more original concepts.
Cautious				
	Quantity and scope	Excessive, limited range, moderate, balance	27	I believe using GenAI within a limited range, such as proofreading my own writing, is acceptable.
	Use and operation	Assistance, proper use, permission	11	I think using GenAI as a learning assistant is good, but relying on it entirely is not advisable.
	Perception and emotion	Unclear, anxiety	5	I still don’t fully understand GenAI, so I have some anxiety, but I also have a desire to master its use.
Negative				
	Thinking and creativity	Think independently, cessation of thinking, boring	40	Our ability to think independently will be lost.
	Ethics and norms	Dishonesty, fairness, rules	9	Presenting GenAI-generated work as one’s own is, in my view, equivalent to plagiarism.
	Uncertainty and constraints	Ambiguity, limitations	3	Accuracy of GenAI is not guaranteed.

## Discussion

Most medical students had used GenAI before university; however, structured learning was limited. Minor differences in perceptions between those with and without learning experience suggest that casual exposure alone may be insufficient to develop a critical understanding of GenAI, highlighting the need for foundational GenAI education in universities [7].

Although the students were interested in learning about GenAI, they remained cautious about using it for assignments, reflecting varied levels of familiarity and trust. While most viewed GenAI positively, some expressed concern that overreliance could hinder creativity and critical thinking.

GenAI dependence may hinder originality and decision-making [8], whereas trust in GenAI may foster motivation and proactive learning [9]. Students’ neutral views suggest uncertainty or low confidence regarding appropriate use, underscoring the need for a balanced education that addresses concerns and fosters critical evaluation skills. This aligns with previous studies highlighting the importance of reforming curricula to integrate GenAI-related competencies, including ethical reasoning, clinical relevance, and communication skills [10].

A limitation of this study was that only first-year students at a single institution were included. Further, the “concern” subscale had relatively low internal consistency, and qualitative analysis relied on a single open-ended item. Finally, multiple comparisons were performed without correction, increasing the risk of type I errors. Nevertheless, this exploratory study aimed to identify trends rather than test specific hypotheses.

In conclusion, our findings support the inclusion of structured GenAI curricula in higher education, suggesting that such programs go beyond technical training to address students’ expectations, values, and concerns.

## Abbreviations

GenAI: generative artificial intelligence
